# Susceptibility Profiles Among *Sporothrix brasiliensis* Genotypes in the Ongoing Zoonotic Sporotrichosis Epidemic in Brazil, Argentina and Paraguay

**DOI:** 10.1007/s11046-026-01113-1

**Published:** 2026-10-15

**Authors:** Vanessa Brito de Souza Rabello, Marcus de Melo Teixeira, Fernando Almeida-Silva, Thiago Brasil de Moura Costa, Andrea Reis Bernardes-Engemann, Melissa Orzechowski Xavier, Rodrigo Almeida-Paes, María Cecilia López-Joffre, Cristina Elena Canteros, Lisandra Serra Damasceno, Isabella Dib Ferreira Gremião, José Guillermo Pereira Brunelli, Wieland Meyer, Rosely Maria Zancopé-Oliveira

**Affiliations:** 1 Laboratório de Micologia, Instituto Nacional de Infectologia Evandro Chagas, Fundação Oswaldo Cruz (Fiocruz), Rio de Janeiro, Brazil; 2 Núcleo de Medicina Tropical, Faculdade de Medicina, Universidade de Brasília (UnB), Brasília, Brazil; 3 Laboratório de Micologia, Faculdade de Medicina, Universidade Federal Do Rio Grande (FAMED-FURG), Rio Grande, Rio Grande do Sul (RS) Brazil; 4 Laboratorio Nacional de Referencia Em Micología Clínica, Departamento de Micología, Instituto Nacional de Enfermedades Infecciosas INEI-ANLIS “ Dr. Carlos G. Malbrán, CABA, Argentina; 5 Hospital São José de Doenças Infecciosas (HSJ), Fortaleza, Ceará Brazil; 6 Faculdade de Medicina, Departamento de Saúde Comunitária, Universidade Federal do Ceará, Fortaleza, Brazil; 7 Laboratório de Pesquisa Clínica Em Dermatozoonoses Em Animais Domésticos, Instituto Nacional de Infectologia Evandro Chagas, Fundação Oswaldo Cruz (Fiocruz), Rio de Janeiro, Brazil; 8 Centro de Especialidades Dermatologicas, Ministerio de Salud Pública y Bienestar Social, San Lorenzo, Paraguay; 9 Westerdijk Fungal Biodiversity Institute, Utrecht, Netherlands

**Keywords:** *Sporothrix brasiliensis*, Genetic variability, Antifungal susceptibility

## Abstract

*Sporothrix brasiliensis* is the predominant etiological agent of sporotrichosis in Brazil and has been progressively expanding its geographic distribution to other countries in South America. The human patients infected with *S*. *brasiliensis* usually respond favorably to antifungal therapy. However, therapeutic failure may occur, and management may be particularly challenging in cases of severe disease. We recently reported that at least seven *S. brasiliensis* genotypes circulate across South America. However, their biological and clinical relevance of this genetic diversity remains poorly understood. In this study, we investigated whether distinct *S*. *brasiliensis* genotypes are associated with reduced susceptibility to the main antifungal agents used in the treatment of human and animal sporotrichosis. A total of 92 clinical, veterinary, and environmental *S. brasiliensis* strains from Brazil, Paraguay and Argentina, genotyping them using a four-gene MLST scheme. Mating type was assessed as well as antifungal susceptibility according to the M38-A2 CLSI guidelines. Results were interpreted according to the established epidemiological cutoff values for *S. brasiliensis*. The isolates are clustered into six distinct genotypes, with mating type showing a strong association with phylogenetic clades. Non-wild type strains were identified across four haplotypes (H1, H2, H3 and H4) for all antifungal drugs tested. These non-wild type strains were isolated from six distinct Brazilian states and were not associated to a specific genetic background. Nevertheless, non-wild-type strains were predominantly detected among feline isolates, most of which originated from Minas Gerais and belonged to haplotype H2. However, because only a limited number of H2 isolates were included in this study, additional isolates from this haplotype are need to confirm this association. Together, these findings suggest that decreased antifungal susceptibility in *S. brasiliensis* is not genotype-restricted but may be influenced by host-related or epidemiological factors, particularly in the context of feline sporotrichosis.

**Supplementary Information:**

The online version contains supplementary material available at https://doi.org/10.1007/s11046-026-01113-1.

## Introduction

Sporotrichosis is an endemic mycosis [1] caused by thermodimorphic fungi of the genus *Sporothrix*. In Brazil, it is the main subcutaneous/implantation mycosis, widely dispersed over the country, with high number of the cases in South and Southeast regions [2, 3]. *Sporothrix brasiliensis* is the main agent of the disease in Brazil and has been attracting attention due to spread [2, 4, 5]. The expansion of the emergence of *S. brasiliensis* in the Northeast region of the country [6–12] and recently in the Midwest [11, 13–15] and North [16, 17] have been described. For instance, the state of Amazonas diagnosed 950 human cases and 2823 animal cases from 2020 to 2023 [16]. Contrary to the previous belief that this species was restricted to Brazil, new cases have already been documented in Argentina, Chile, Uruguay and Paraguay [18–25].

The disease usually occurs after a traumatic inoculation of materials containing *Sporothrix* spp., such as decaying vegetation and wood, through the skin [26, 27]. In Brazil, sporotrichosis is highly associated with zoonotic transmission by scratches or bites of infected cats [2, 3, 21] with *S. brasiliensis* being the main species isolated in the country and highly associated with zoonotic cases of the mycosis [2, 3, 21, 28].

Several clinical forms of sporotrichosis have been described, but the majority of patients develop the lymphocutaneous form followed by the fixed cutaneous form [2, 27]. Moreover, systemic manifestations involving the osteoarticular system, lungs, and the central nervous system (CNS) have been increasingly recognized [29–33], posing significant therapeutic challenges [31, 34–36]. In addition to classical cutaneous forms, *S. brasiliensis* infection has been associated with atypical immunoreactive manifestations, including erythema multiforme, erythema nodosum, and Sweet syndrome [37–39].

Currently, itraconazole is the drug of choice for sporotrichosis treatment. Terbinafine and potassium iodide are considered alternative options for managing localized forms of the disease [27]. Amphotericin B is indicated for severe cases or for patients who fail to respond to treatment with other antifungal therapies [27, 36]. Most patients infected with *S*. *brasiliensis* respond favorably to standard antifungal therapy [40]. However, therapeutic failures have been increasingly reported, raising concerns about reduced antifungal susceptibility in clinical settings, particularly in the context of expanding zoonotic transmission [41–44].

Method-dependent and species-specific epidemiological cutoff values (ECVs) enable the differentiation between wild-type and non-wild-type isolates. Wild-type isolates lack phenotypically detectable acquired resistance mechanisms and are expected to exhibit the intrinsic susceptibility profile of the species, whereas non-wild-type isolates have MIC values above the established ECVs, suggesting reduced susceptibility associated with acquired or emerging resistance mechanisms. Although ECVs do not predict the clinical outcome of antifungal therapy, they identify isolates that are less likely to respond to a given antifungal agent.

ECVs have been established for *S. brasiliensis* and *S. schenckii* based on susceptibility data generated in a multicenter study [45]. Non-wild-type isolates exhibit MIC values exceeding the corresponding ECVs and are therefore more likely to show reduced susceptibility to the antifungal agents tested, although the underlying molecular mechanisms remain poorly understood [46]. Surveillance of antifungal susceptibility is essential to identify putative non-wild-type *Sporothrix* isolates, as infections caused by these strains may require prolonged treatment or higher antifungal doses [47].

Genetic diversity may contribute to variability in antifungal susceptibility. Under selective pressure, *S. brasiliensis* may undergo chromosomal polymorphisms, including changes in chromosome number and size, which could facilitate the emergence of clones with reduced antifungal susceptibility [48]. In addition, single nucleotide polymorphisms and chromosomal copy number variations have been proposed as potential mechanisms underlying decreased susceptibility. This hypothesis is supported by the identification of non-wild-type *S. brasiliensis* isolates exhibiting elevated MIC values for itraconazole and amphotericin B in vitro [49].

The aim of the present study was to analyze the susceptibility profiles of *S*. *brasiliensis* genotypes against the main drugs used in the sporotrichosis treatment: itraconazole, terbinafine and amphotericin B, by determining their MICs and associating non-wild type strains to genotypes and geographic regions through South America.

## Material and Methods

### Fungal Strains and Genotyping

A total of 92 *S*. *brasiliensis* strains from human patients (n = 39), cats (n = 49), dogs (n = 2) and from the environment (n = 2) obtained from different geographic regions throughout Brazil, Paraguay and Argentina were included in this study (Online Resource 1). DNA was extracted from the yeast phase as described previously (Ferrer et al., 2001). The strains were identified as *S*. *brasiliensis* using species-specific polymerase chain reaction (PCR) assay as described by Rodrigues et al. [50]. Then, the 92 *S*. *brasiliensis* strains were genotyped by Multi locus sequence typing (MLST) according to Rabello et al. [51], using the following primer pairs: Aspartate aminotransferase (*AST-3)* (F-CAAAATGCACGAGGACTGCC) and (R-GCACTGACACAACGCTCAAG), Glycerol-3-phosphate dehydrogenase (*GPD1-2)* (F- CATCATCCTGCCCGGTAACA), (R-GGACTCGTTACGAACACCGT), Cytosolic phospholipase A2 (*cPLA2-1)* (F- ATCTGCTTCTTGCGCTCGTA), (R-CAATGGCAAACAGGGCTCAC) and an uncharacterized protein (SPBR_05954) (F- GGAGAGGCCAATAGCAGCTT), (R- CGGGAGAACCGCTACAAGAA). The PCR amplicons were purified using the QIAquick PCR Purification Kit (Qiagen) and Sanger-sequenced was done using the FIOCRUZ Technological Platforms. The sequences were manually edited with the software Sequencher version 4.9 (Gene Codes Corporation, Michigan, USA) and aligned by MEGA version 6. Phylogenetic analyses were carried out using Maximum Likelihood method with the Tamura-Nei nucleotide substitution model in MEGA 6 software. Indices of branch support were generated by 1,000 bootstraps and were added next to the branches. The genetic diversity rates were calculated using the software DNAsp v5.0 [52]. The haplotype network was built with the software Network 10.2.0.0 using the Median-joining networks method [53].

## Mating Type Locus Characterization

The mating type loci of *S. brasiliensis* was determined by PCR using the MAT1-1-1(Mat1_1_1F,AAGCACGCCAGTTTCATTCT; Mat1_1_1R,CACCAACGAGCATCTCATGT), and MAT1-1-2(Mat1_2F,GATCTCAACGGCCATCTTGT;Mat1_2R, GCTACATACTTCCGCCCTGA) according to the methodology described by Teixeira et al. [54], modified. The PCR reaction to detect the mating-type (MAT) gene were performed in a final volume of 25 μL, including Buffer 1× PCR, 0.2 mM dNTPs, 1.5 mM MgCl2, 1.0 U/μL Taq Polymerase (Invitrogen, Brazil), 0.5 μL of each *primer* (10 µM/μL) and 4 μL DNA (25 ng/μL). The amplification conditions were: 35 cycles of initial denaturation at 95 °C for 5 min, denaturation at 94 °C for 30 s, annealing at 60 °C for 45 s, extension at 72 °C for 30 s, and final extension at 72 °C for 5 min.

## Antifungal Susceptibility Assay

The broth microdilution reference method was performed according the M38-A2 CLSI guideline [55] in 96-well microplates. Three drugs were tested: itraconazole (ITZ), terbinafine (TRB) and amphotericin B (AMB). The tests were performed at least twice on different days. *Aspergillus fumigatus* (ATCC 204305) and *Aspergillus flavus* strains (ATCC 204304) were used as controls.

Stock solutions of ITZ, TRB and AMB (Sigma-Aldrich Corporation, San Luis, MO, USA) were prepared using dimethyl sulfoxide (DMSO) at 1600 μg/mL. From the stock solutions, two-fold dilutions were performed in RPMI-1640 with phenol red, without sodium bicarbonate buffered with 3-(N-Morpholino) propanesulfonic acid (MOPS), pH 7.0 to obtain the antifungal final concentrations, which ranged from 0.03 to 16 μg/mL. A volume of 100 μL of RPMI-1640 buffered with MOPS and containing serial dilutions of the antifungal agents was added to each well of the microtiter plates. An inoculum of 1–5×10^4^ conidia/mL was prepared in sterile saline solution following incubation of each strain on potato dextrose agar (PDA) for seven days at 35 °C. Fungal conidia suspension (100 μL) diluted 1:50 in RPMI-1640 buffered with MOPS supplemented with 4% glucose were then added to each well-containing the drug dilutions. Positive controls consisted of 100 μL of conidia suspension prepared as above without any antifungal drug. Negative controls consisted of 200 μL of RPMI 1640 medium without any antifungal drug and fungal inoculum. Plates were incubated at 35 °C for 48–72 h and the MIC was determined visual inspection as described [55]. For ITR and AMB, the MIC endpoints were the lowest concentrations that completely inhibited fungal growth. For TRB, it was the lowest concentration that resulted in at least an 80% reduction in growth relative to the positive control without the antifungal drug [45].

ECVs were based on data from an international study evaluating antifungal susceptibility in several *Sporothrix* species. According to this study *S*. *brasiliensis* strains with MIC values for ITR above 2.0 μg/mL, TRB above 0.12 μg/mL and for AMB above 4.0 μg/mL were classified as non-wild type strains [45]. The determination of the frequency of MIC values was calculated with the Microsoft Excel (2016) software. Analysis of the proportion of non-wild type between groups using Fisher’s exact test. To evaluate whether mating type frequencies deviated from the expected 1:1 ratio, exact binomial tests were performed for each genetic group. Statistical significance was set at *p* < 0.05 (software R Core Team 2025, version 4.5.1).

## Results

**Fig. 1:**
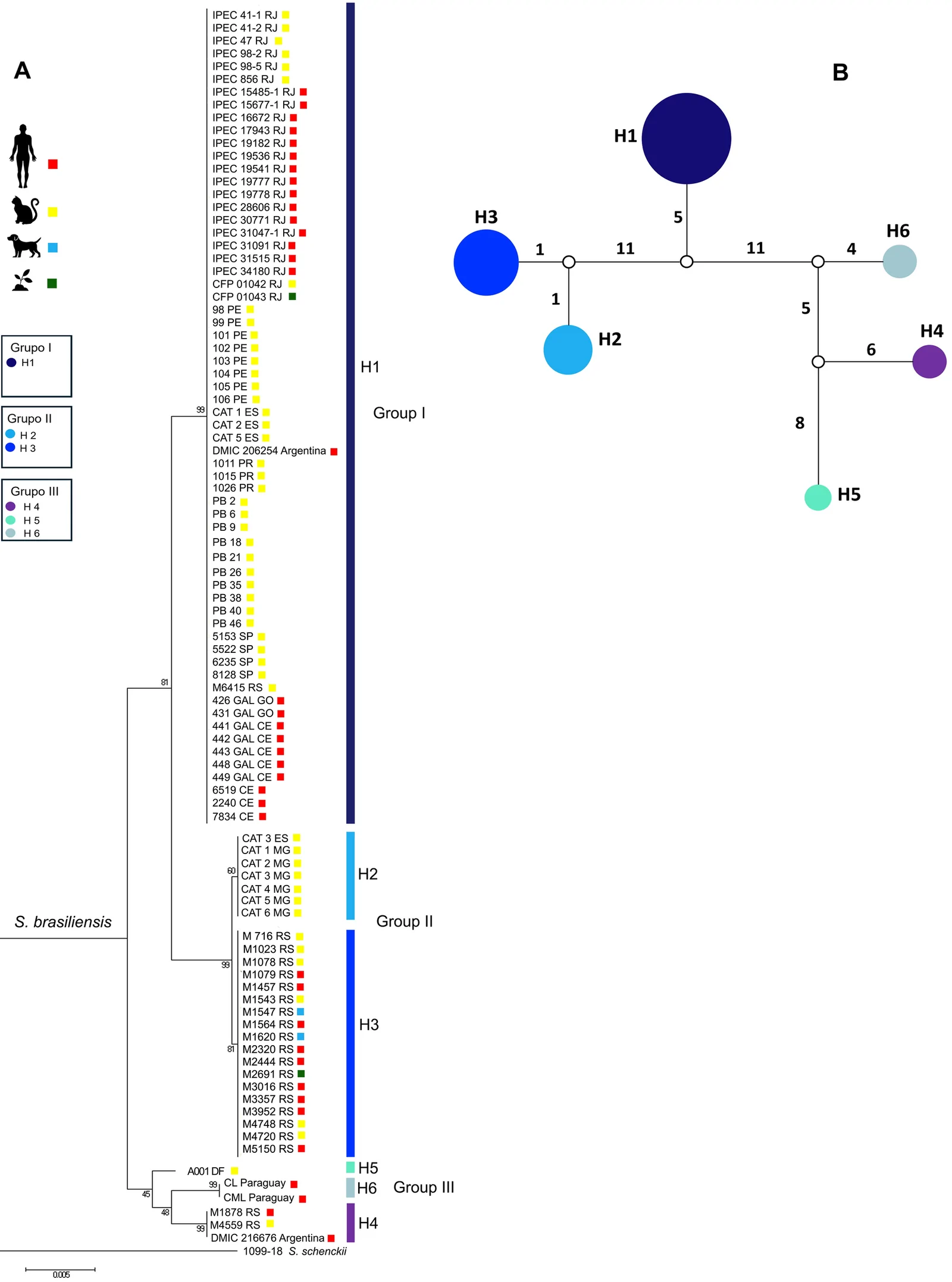
The **a** Genetic relationships of *S. brasiliensis* by maximum likelihood analysis using the four concatenated multilocus sequences: AST-3, cPLA2-1, GPD1-2, and SPBR_05954. Indices of support based on 1000 bootstrap replications. **b** Haplotype network of *S. brasiliensis* by median-joining based on the four multilocus sequences

Based on MLST analysis, we identified at least three distinct *S*. *brasiliensis* genotypes in Brazil (Group I, Group II, Group III). Group I include most of the isolates from the Southeast (n = 29), all isolates from Northeast (n = 25), some isolates from South, Paraná state (n = 3), and one isolate from Rio Grande do Sul, besides one from Argentina, and from Midwestern (n = 2). Most of the strains from state of Rio Grande do Sul (n = 18), situated on South were clustered into Group II, along with all isolates from Minas Gerais (n = 6) and one isolate from Espírito Santo on Southeast region. The Group III were also diverse including one isolate from Federal District and two from Rio Grande do Sul, as well the isolates from Paraguay (n = 2), and one isolate from Argentina (Fig. 1a). Among these groups, the strains were classified into six haplotypes described for *S*. *brasiliensis* (Fig. 1b).

**Fig. 2:**
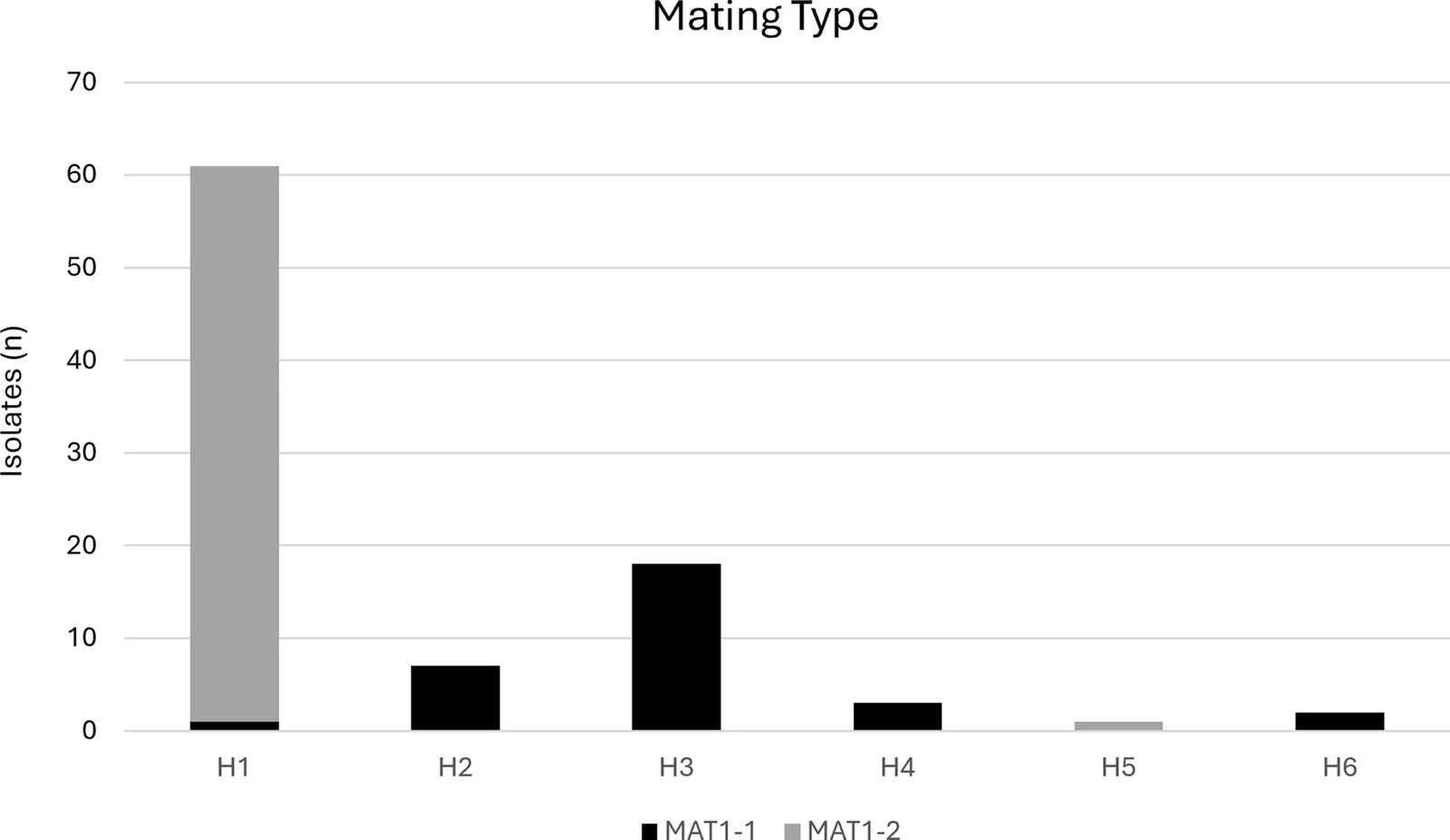
Mating type distribution according to the six haplotypes (H1, H2, H3, H4, H5 and H6)

Mating type was associated with groups, 66% of *S. brasiliensis* isolates carrying MAT1-2 allele, most of them in the haplotype H1 (Fig. 2), MAT1-2 was strongly predominant in the haplotype H1 with significant deviation from the expected distribution (*p* < 0.0001), just one isolate in the haplotype H1 from Rio Grande do Sul carried MAT1-1 allele. Likewise, MAT1-1 was associated with haplotype H2 and H3 (*p* 0.01), (*p* < 0.0001) respectively. In other haplotypes mating type did not significantly differ from the expected ratio (*p* > 0.5), mainly due to small count of isolates. The distribution of mating types within each genetic group was also assessed to determine whether it deviated from the expected 1:1 ratio using exact binomial tests. A significant deviation from the expected ratio was observed in groups I and II. In group I, MAT1-2 was strongly predominant (MAT1-2 n = 60, MAT1-1 n = 1), resulting in a highly significant deviation from the expected distribution (*p* < 0.0001). Likewise, group II exhibited only MAT1-1 isolates (MAT1-1 n = 25, MAT1-2 n = 0), also showing a significant deviation from the expected 1:1 ratio (*p* < 0.0001). In contrast, the mating type distribution in group III (MAT1-1 n = 5, MAT1-2 n = 1) did not significantly differ from the expected ratio (*p* = 0.22).

Overall, there is a strong predominance of a single mating type in some groups. This pattern may suggest population structure, clonal expansion, and potentially limited sexual recombination in groups I and II. Conversely, the more balanced mating type distribution observed in group III may be consistent with higher genetic diversity and the potential for ongoing sexual recombination within this group.

The in vitro antifungal susceptibility assay to three antifungal drugs, terbinafine showed the lowest and amphotericin B the highest MIC values. Table 1 summarizes the in vitro antifungal susceptibility of these 92 *S*. *brasiliensis* strains. To date, there are no defined clinical breakpoints for *Sporothrix* spp.*,* therefore we cannot classify the isolates as susceptible or resistant. However, according to the ECVs (Espinel-Ingroff et al., 2017), 11 strains (11.95%) were classified as non-wild type, three isolated from humans and eight from cats (Table 2). We identified non-wild type strains to ITR (n = 10), TER (n = 6) and to AMB (n = 6). Some strains (n = 5) were non-wild type to all antifungal drugs, both of them isolated from cats.

**Table 1:** Minimum inhibitory concentrations (μg/mL) of 92 *S*. *brasiliensis* strains tested against the three main antifungal drugs used for sporotrichosis treatment

Antifungal drug	Range		MIC 50	MIC 90	Geometric mean
	Minimum	Maximum			
Itraconazole	0.12	> 8	0.5	1	0.7
Terbinafine	0.015	0.5	0.06	0.12	0.06
Amphotericin B	0.25	> 8	1	4	1.3

**Table 2:** Summary of minimum inhibitory concentration (μg/mL) of *S*. *brasiliensis* strains tested in this study

Antifungal drug	Human (n = 39)	Cat (n = 49)	Dog (n = 2)	Environment (n = 2)
Itraconazole	0.12– > 8	0.12– > 8	0.5	0.25
Terbinafine	0.015–0.12	0.015– 0.5	0.03–0.06	0.03–0.06
Amphotericin B	0.25–8	0.5– > 8	1	1
Non-wild type (n)	3	8	0	0

**Fig. 3:**
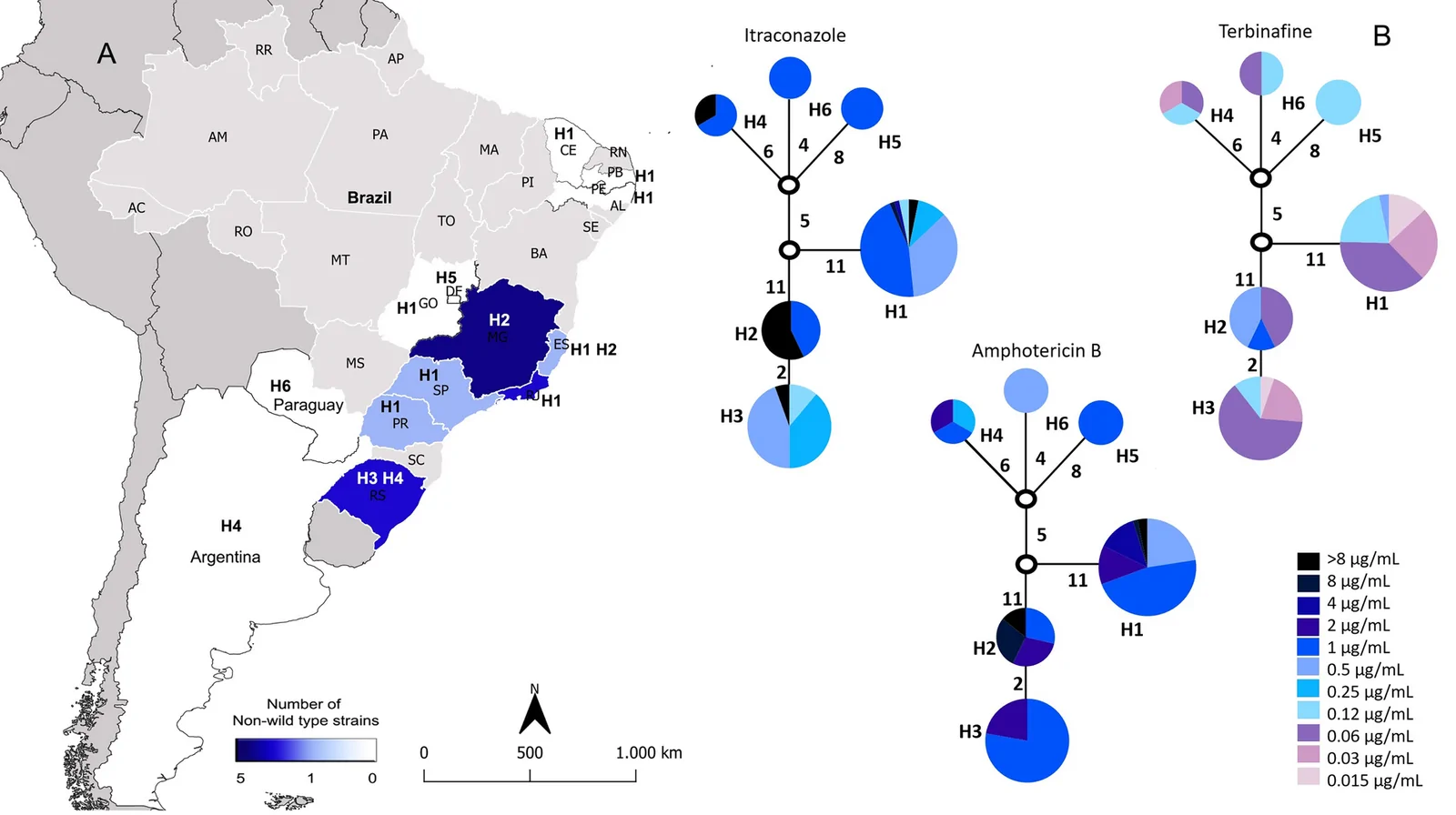
The **a** Geographic region of non-wild type* S*. *brasiliensis* strains. **b** Susceptibility profiles of *S*. *brasiliensis* genotype. Haplotype network of *S*. *brasiliensis* by Median-joining based on the four multilocus sequences: AST-3, cPLA2-1, GPD1-1 and SPBR_05954

The non-wild type strains are distributed within four haplotypes, H1, H2, H3 and H4 (Table 3), along with isolates from Rio de Janeiro, Rio Grande do Sul, São Paulo, Paraná, Minas Gerais e Espírito Santo (Table 4 and Fig. 3a and b).

**Table 3:** Minimum inhibitory concentration (μg/mL) of *S*. *brasiliensis* genotypes

Haplotype	Isolates (n)	Non-wild type (n)	Itraconazole	Terbinafine	Amphotericin B
H1	61	4	0.12– > 8	0.015–0.5	0.5– > 8
H2	7	5	1– > 8	0.06–0.5	1– > 8
H3	18	1	0.12– > 8	0.03–0.12	1–2
H4	3	1	1– > 8	0.03–0.12	0.25–2
H5	1	-	1	0.12	1
H6	2	-	1	0.06–0.12	0.5

**Table 4:** Minimum inhibitory concentration (μg/mL) of *S*. *brasiliensis* by geographic region

MIC Range of Region					
Geographic Region	Isolates (n)	Non-wild type (n)	Itraconazole	Terbinafine	Amphotericin B
Rio de Janeiro	23	2	0.12– > 8	0.015–0.12	0.5– > 8
Rio Grande do Sul	21	2	0.12– > 8	0.03–0.12	1–2
Paraíba	10	–	0.5–1	0.03–0.12	1–4
Pernambuco	7	–	0.25–1	0.03–0.06	1–4
Ceará	8	–	0.5–1	0.03–0.06	0.5–1
Espírito Santo	3	1	1	0.06–0.5	1
Minas Gerais	6	4	1– > 8	0.06–0.5	1– > 8
São Paulo	4	1	0.25–> 8	0.06–0.5	0.5– > 8
Paraná	3	1	1– > 8	0.015–0.5	1– > 8
Federal District	1	–	1	0.12	1
Goiás	2	–	0.5–1	0.06	0.5–1
Paraguay	2	–	1	0.06–0.12	0.5
Argentina	2	–	1	0.03	0.25–1

Comparison between haplotype H2 and the other haplotypes (H1, H3, H4, H5, H6) indicated a significantly higher proportion of non-wild type isolates in H2 (71.4%, 5/7) compared to 7.0% (6/85) among the others. Fisher’s exact test demonstrated a significant association (*p* = 0.0001752), with an elevated odds ratio 30.01 (95% CI 3.98–376.19) indicating that isolates belonging to haplotype H2 were more likely to be non-wild type.

## Discussion

Recently we reported an important advance in the *S. brasiliensis* knowledge through the development of a novel multilocus sequence typing (MLST) for genotyping of *S*. *brasiliensis* isolates. This method substantially increases the discriminatory.

power and provides new contributions to epidemiological studies of this human and animal pathogen in Brazil and other countries [51].

The previous results led us to question whether there might be differences in antifungal susceptibility among the different genotypes evaluated. In this study, was included the molecular characteristics of the isolates genotyped and their in vitro susceptibility to three antifungal drugs. This study highlights that there are at least three distinct *S*. *brasiliensis* genotypes circulating in Brazil (Group I, Group II, Group III). Moreover, there are genetically distinct genotypes identified from Paraguay (haplotype H6) and one isolate from Argentina (haplotype H4). This may indicate the existence of a separate *S. brasiliensis* population in these countries.

The isolate DMIC 206254 from Argentina is highly related to the main Group I, haplotype H1 along with most of the Brazilian isolates, and the isolate DMIC 216676, also from Argentina grouped into Group III, haplotype H4 close to same isolates from Rio Grande do Sul, Brazil. These data suggest that both Argentinian isolates could be introduced from Brazil to Argentina through a sick cat or *S. brasiliensis* is also endemic species in this country.

It is possible that the introduction of *S. brasiliensis* might be facilitated by the movement of sick or colonized cats. Fernandez et al., 2024 using short tandem repeat (STR) genotyping, showed that two cases from Argentina related to zoonotic transmission had identical genotypes, which likely originate from the same source, sick cats. Moreover, a large urban outbreak of feline sporotrichosis caused by *S. brasiliensis* was described in Puerto Iguazú, Argentina which borders Brazil [25]. This could reflect transmission dynamics like the zoonotic epidemic in Brazil. That started in the late 1990s in Rio de Janeiro state [56, 57], and *S. brasiliensis* are circulating in Argentina also since at least 1986 [58].

Nevertheless, the emergence of the zoonotic sporotrichosis in Argentina is more recent than in Brazil. The first cat-transmitted human case of sporotrichosis in Brazil date back to 1955 in São Paulo [59]. With this, it may have been either an expansion of a Brazilian genotype to neighboring countries, or that *S. brasiliensis* harbors distinct genotypes native to each country.

The zoonotic transmission should cause an alert, since sick cats could transmit the disease by *S. brasiliensis* to region, which have never supposed to happen. This fact was recently demonstrated, the first *S. brasiliensis* cases reported in the United Kingdom, associated with an infected cat imported from Brazil [60]. This fungus can quickly be dispersed, causing a severe public health problem when it is introduced into a cat population. Thus, the development of public policies is essential to curb the expansion of *S. brasiliensis* and feline-transmitted sporotrichosis, and public health officials should coordinate One Health actions. As demonstrated in a previous study, cat’s mass treatment can reduce substantially the disease prevalence, and this effect is potentialized when combined with neutering [61].

Regarding the genotyping method, we previously highlighted that there were some groups which clustered according to their geographic origins, the group II along with haplotype 2 and 3 included mainly isolates from Minas Gerais and Rio Grande do Sul respectively [51], herein validated and the phylogenetic tree shows three major monophyletic groups in Brazil. Moreover, group III included both isolates from Paraguay (haplotype 6), one from Distrito Federal (haplotype 5), two from Rio Grande do Sul and one from Argentina (haplotype 4) showing great genetic variation in *S. brasiliensis*.

Previous findings showed intraspecific variability among *S. brasiliensis* isolates emerging independently in different regions of Brazil [5, 11, 13, 62]. Clear evidence has shown that *S. brasiliensis* genotypes circulating in animals from Minas Gerais are distinct from those from Rio de Janeiro [5, 62]. Our results support this observation, Group II included all isolates from Minas Gerais (H2), and the majority from Rio Grande do Sul (H3), that are separated from those Rio de Janeiro isolates. Furthermore, isolates from Minas Gerais and Rio Grande do Sul predominantly carried the *MAT1-1* idiomorph, while genotypes from Rio de Janeiro and several other Brazilian states were mainly associated with *MAT1-2*.

It is important to emphasize that genetic variability can be assessed using different methods. Approaches such as STR genotyping, MLST, and amplified fragment length polymorphism (AFLP) can used as whole-genome sequencing (WGS) single nucleotide polymorphism (SNP) analysis in *S. brasiliensis* [11, 24, 51]. The major strength of MLST in relation to other methods applied to typing *S. brasiliensis* it is easy to perform in any laboratory with limited budget and/or infrastructure, which unfortunately is common in areas where *S. brasiliensis* is endemic, mainly in Brazil providing more reliable data on the genetic diversity of this fungus. However, different *S. brasiliensis* clades can be found when these methods are used in isolates from different regions. One important limitation of the current work is the number of isolates, which might not accurately reflect the *S. brasiliensis* population in Brazil, Paraguay and Argentina. That is the limitation of the present and of many other studies, independent of the method used.

Regarding to antifungal susceptibility, some *S*. *brasiliensis* strains were classified as non-wild type in our study to the main antifungal drugs itraconazole, terbinafine and amphotericin B. The non-wild type strains were distributed in different haplotypes, but mainly in Southeast and South of Brazil. Zoonotic transmission in Southeastern Brazil started between the late 1950s in São Paulo [59] and 1998 cases of sporotrichosis began to emerge in cats in Rio de Janeiro with a large number of cases. Around 2015 outbreaks taking place in other Southeast Brazilian states, as Minas Gerais and Espírito Santo, and in the South of Brazil, with many cases in Rio Grande do Sul [2, 63]. All along these years was marked by the low frequency of zoonotic transmission in Northeast, North and Midwest Brazil [2, 63]. Therefore, the recent sporotrichosis epidemic in these three last Brazil regions could explain low emergence non-wild type strains compared with them that regions with many human and animal cases in the last 20 years.

It is important to note that most of the non-wild type strains were isolated from cats. Furthermore, the haplotype H2 harbored most of the non-wild type strains, although four isolates with haplotype H1 and one with haplotype H3 and H4 respectively also showed high MIC values. The haplotype H2 included all isolates from Minas Gerais (n = 6) e and one isolate from Espírito Santo, that showed higher MIC to terbinafine. Among the six isolates from Minas Gerais, three of them showed high MIC to all antifungal classes and one isolated had high MIC value to itraconazole (> 8 μg/mL).

The genotype H2 showed a significantly higher frequency of non-wild type isolates compared with the other genotypes evaluated. However, the wide confidence interval may reflect the imprecision associated with the small number of H2 isolates included in this study. Therefore, these findings should be validated using a larger number of isolates representing haplotype H2. Moreover, it should not emphasize that the haplotype H2 is more resistant to antifungal drugs, since it was not analyzed the genetic mutation related to the genes resistance as well as reported by prior studies [49]. Some studies analyzing the whole-genome sequencing did not identify an association between MIC values to itraconazole and phylogenetic structure [64], since non-wild type strains to itraconazole clustered with susceptible ones, which was also demonstrated in our study.

We would like to emphasize that the non-wild type isolates are circulating among sick cats, since most of these strains were obtained from cats, as well as all isolates from haplotype H2 were isolated from sick cats. Therefore, this zoonotic transmission may favor the selection of non-wild type strains. Collectively, these findings indicate that reduced antifungal susceptibility in *S. brasiliensis* is not genotype-restricted but is likely shaped by host-related and epidemiological factors, particularly within the context of the ongoing feline-associated zoonotic epidemic.

Although a previous study did not observe significant differences in minimal inhibitory concentration values between cat and human isolates [4], cats are the most susceptible hosts to *S. brasiliensis*. Consequently, non-wild type strains can be readily transmitted by infected cats, which may act as carriers for resistant phenotypes/genotypes throught rapid clonal expansion. Moreover, the thermal resistance of this species to cat's body temperature, around 39 °C can partially explain the success of infection, and the feline inflammatory immune response to combat an advanced sporotrichosis infection is less efficient, and the fungal load in the infection site is high [21], which makes cat’s treatment a challenge [65]. Besides, the cats’ behavior generally outdoors, they scratch the vegetation, which facilitates sporotrichosis infection and due to their instinct, fights between males, which promotes the quick disease transmission between them.

Therefore, the emergence of these non-wild type strains could increase the number of sporotrichosis cases in Brazil, making treatment more challenging, since patients infected with those strains usually present adverse outcomes [47]. However, clinical data were not available in this study, preventing the evaluation of potential correlations between clinical outcomes, in vitro antifungal susceptibility, and the genotypes identified. Although, this study highlights the genetic diverse among *S*. *brasiliensis* from different Brazilian states and other South American countries. Moreover, antifungal susceptibility data may contribute to understand the potential emergence of antifungal resistance in the context of zoonotic sporotrichosis.

## Supplementary Information

Below is the link to the electronic supplementary material.

Supplementary file1 (DOCX 44 kb)

Supplementary file2 (DOCX 43 kb)

## Data Availability

The original contributions presented in the study are included in the article or supplementary information file. Further inquiries can be directed to the corresponding author.
