# Supplementary material for: Susceptibility Profiles Among Sporothrix brasiliensis Genotypes in the Ongoing Zoonotic Sporotrichosis Epidemic in Brazil, Argentina and Paraguay
Source: Mycopathologia. 2026 Oct 15;191(6):89. doi: 10.1007/s11046-026-01113-1 (PMC13672573; doi:10.1007/s11046-026-01113-1)
Supplement: Supplementary file 1 [file 11046_2026_1113_MOESM1_ESM.docx]

Susceptibility Profiles Among *Sporothrix brasiliensis* Genotypes in the Ongoing Zoonotic Sporotrichosis Epidemic in Brazil, Argentina, and Paraguay

Mycopathologia

Vanessa Brito de Souza Rabello^1*^ ([0000-0002-9318-6240](https://orcid.org/0000-0002-9318-6240)), Marcus de Mello Teixeira^2^ ([0000-0002-3330-3847](https://orcid.org/0000-0002-3330-3847)), Fernando Almeida-Silva^1^ ([0000-0002-7378-1990](https://orcid.org/0000-0002-7378-1990)), Thiago Brasil de Moura Costa^1^ ([0009-0004-2448-3198](https://orcid.org/0009-0004-2448-3198)), Andrea Reis Bernardes-Engemann^1^ ([0000-0002-4997-8734](https://orcid.org/0000-0002-4997-8734)), Melissa Orzechowski Xavier^3^ ([0000-0002-3883-0080](https://orcid.org/0000-0002-3883-0080)), Rodrigo Almeida-Paes^1^ ([0000-0002-6639-6058](https://orcid.org/0000-0002-6639-6058)), María Cecilia López-Joffre^4^ ([0009-0006-1479-0615](https://orcid.org/0009-0006-1479-0615)), Cristina Elena Canteros^4^ ([0000-0002-0955-9340](https://orcid.org/0000-0002-0955-9340)), Lisandra Serra Damasceno^5^ ([0000-0003-1425-5912](https://orcid.org/0000-0003-1425-5912)), Isabella Dib Ferreira Gremião^6^ ([0000-0001-8428-7616](https://orcid.org/0000-0001-8428-7616)), José Guillermo Pereira Brunelli^7^ ([0000-0001-9660-4156](https://orcid.org/0000-0001-9660-4156)), Wieland Meyer^8^ ([0000-0001-9933-8340](https://orcid.org/0000-0001-9933-8340)), Rosely Maria Zancopé-Oliveira^1^ ([0000-0001-7619-1921](https://orcid.org/0000-0001-7619-1921))

1- Laboratório de Micologia, Instituto Nacional de Infectologia Evandro Chagas, Fundação Oswaldo Cruz, Rio de Janeiro, Brazil.

2- Núcleo de Medicina Tropical, Faculdade de Medicina, Universidade de Brasília (UnB), Brasília, Brazil.

3- Laboratório de Micologia, Faculdade de Medicina, Universidade Federal do Rio Grande (FAMED-FURG), Rio Grande do Sul (RS), Brazil.

4- Laboratorio Nacional de Referência em Micologia Clínica, Departamento de Micología. Instituto Nacional de Enfermedades Infecciosas INEI-ANLIS “Dr. Carlos G. Malbrán, CABA, Argentina.

5- Hospital São José de Doenças Infecciosas (HSJ), Fortaleza, Ceará, Brazil.

6- Laboratório de Pesquisa Clínica em Dermatozoonoses em Animais Domésticos, Instituto Nacional de Infectologia Evandro Chagas, Fundação Oswaldo Cruz (Fiocruz), Rio de Janeiro, Brazil.

7- Centro de Especialidades Dermatologicas, Ministerio de Salud Pública y Bienestar Social, Paraguay.

8- Westerdijk Fungal Biodiversity Institute, Utrecht, Netherlands.

* Corresponding authors: [vanessabritorabello@gmail.com](mailto:vanessa.brito@ini.fiocruz.br) (Rabello, V.B.S^1^)

**Table Online Resource 1.** List of fungal strains studied, indicating their strain number, isolation source, geographical location, year of isolation and GenBank accession numbers.

| Isolates | Sources | Geographic origin | Isolation year | AST 3SET | GPD1 2SET | CPLA 2 1SET | SPBR_05954 |
| --- | --- | --- | --- | --- | --- | --- | --- |
| IPEC 41-1 | Cat | Rio de Janeiro | 1999 | ON892956 | OP388696 | ON892857 | OR495608 |
| IPEC 41-2 | Cat | Rio de Janeiro | 1999 | ON892957 | OP388697 | ON892858 | OR495609 |
| IPEC 47 | Cat | Rio de Janeiro | 1999 | ON892958 | OP388698 | ON892859 | OR495610 |
| CFP 01256  (IPEC 98-2) | Cat | Rio de Janeiro | 1999 | ON892959 | OP388699 | ON892860 | OR495611 |
| CFP 01257  (IPEC 98-5) | Cat | Rio de Janeiro | 1999 | ON892960 | OP388700 | ON892861 | OR495612 |
| IPEC 856 | Cat | Rio de Janeiro | 2001 | ON892961 | OP388701 | ON892862 | OR495613 |
| CFP 01254  (IPEC 15485-1) | Human | Rio de Janeiro | 1998 | ON892962 | OP388702 | ON892863 | OR495614 |
| IPEC 15677-1 | Human | Rio de Janeiro | 1999 | ON892963 | OP388703 | ON892864 | OR495615 |
| IPEC 16672 | Human | Rio de Janeiro | 1999 | ON892965 | OP388705 | ON892866 | OR495616 |
| IPEC 17943 | Human | Rio de Janeiro | 2000 | ON892966 | OP388706 | ON892867 | OR495617 |
| CFP 01255  (IPEC 19182) | Human | Rio de Janeiro | 2001 | ON892967 | OP388707 | ON892868 | OR495618 |
| CFP 01022  (IPEC 19536) | Human | Rio de Janeiro | 2001 | ON892968 | OP388708 | ON892869 | OR495619 |
| IPEC 19541 | Human | Rio de Janeiro | 2001 | ON892969 | OP388709 | ON892870 | OR495620 |
| IPEC 19777 | Human | Rio de Janeiro | 2001 | ON892970 | OP388710 | ON892871 | OR495621 |
| IPEC 19778 | Human | Rio de Janeiro | 2001 | ON892971 | OP388711 | ON892872 | OR495622 |
| IPEC 28606 | Human | Rio de Janeiro | 2005 | ON892977 | OP388717 | ON892878 | OR495628 |
| IPEC 30771 | Human | Rio de Janeiro | 2006 | ON892972 | OP388712 | ON892873 | OR495623 |
| IPEC 31047-1 | Human | Rio de Janeiro | 2006 | ON892973 | OP388713 | ON892874 | OR495624 |
| IPEC 31091 | Human | Rio de Janeiro | 2007 | ON892975 | OP388714 | ON892875 | OR495625 |
| IPEC 31515 | Human | Rio de Janeiro | 2007 | ON892974 | OP388715 | ON892876 | OR495626 |
| IPEC 34180 | Human | Rio de Janeiro | 2008 | ON892976 | OP388716 | ON892877 | OR495627 |
| CFP 01043 | Environment | Rio de Janeiro | 2020 | ON892978 | OP388718 | ON892879 | OR495629 |
| CFP 01042 | Cat | Rio de Janeiro | 2017 | ON892979 | OP388719 | ON892880 | OR495630 |
| M716 | Cat | Rio Grande do Sul | 2012 | ON893019 | OP388764 | ON892895 | OR495645 |
| M1023 | Cat | Rio Grande do Sul | 2012 | ON893022 | OP388765 | ON892896 | OR495646 |
| M1078 | Cat | Rio Grande do Sul | 2012 | ON893020 | OP388766 | ON892897 | OR495647 |
| M1079 | Human | Rio Grande do Sul | 2012 | ON893021 | OP388767 | ON892898 | OR495648 |
| M1457 | Human | Rio Grande do Sul | 2013 | ON893023 | OP388768 | ON892899 | OR495649 |
| M1543 | Cat | Rio Grande do Sul | 2013 | ON893024 | OP388769 | ON892900 | OR495650 |
| M1547 | Dog | Rio Grande do Sul | 2013 | ON893025 | OP388770 | ON892901 | OR495651 |
| M1564 | Humano | Rio Grande do Sul | 2013 | ON893026 | OP388771 | ON892902 | OR495652 |
| M1620 | Dog | Rio Grande do Sul | 2013 | ON893027 | OP388772 | ON892903 | OR495653 |
| M1878 | Human | Rio Grande do Sul | 2014 | ON892951 | OP388785 | ON892944 | OR495654 |
| M2320 | Human | Rio Grande do Sul | 2014 | ON893028 | OP388773 | ON892904 | OR495655 |
| M2444 | Human | Rio Grande do Sul | 2015 | ON893029 | OP388774 | ON892905 | OR495656 |
| M2691 | Environment | Rio Grande do Sul | 2015 | ON893030 | OP388775 | ON892906 | OR495657 |
| M3016 | Human | Rio Grande do Sul | 2015 | ON893031 | OP388776 | ON892907 | OR495658 |
| M3357 | Human | Rio Grande do Sul | 2016 | ON893033 | OP388777 | ON892908 | OR495659 |
| M3952 | Human | Rio Grande do Sul | 2017 | ON893032 | OP388778 | ON892909 | OR495660 |
| Isolates | **Sources** | **Geographic origin** | **Isolation year** | **AST 3SET** | **GPD1 2SET** | **CPLA 2 1SET** | **SPBR_05954** |
| M4559 | Cat | Rio Grande do Sul | 2018 | ON892952 | OP388786 | ON892945 | OR495662 |
| M4720 | Cat | Rio Grande do Sul | 2018 | ON893034 | OP388779 | ON892910 | OR495661 |
| M4748 | Cat | Rio Grande do Sul | 2018 | ON893035 | OP388780 | ON892911 | OR495663 |
| M5150 | Human | Rio Grande do Sul | 2019 | ON893036 | OP388781 | ON892912 | OR495664 |
| M6415 | Cat | Rio Grande do Sul | 2021 | ON892993 | OP388733 | ON892913 | OR495665 |
| 1011 PR | Cat | Paraná | 2020 | ON892994 | OP388734 | ON892914 | OR495666 |
| 1015 PR | Cat | Paraná | 2020 | ON892995 | OP388735 | ON892915 | OR495667 |
| 1026 PR | Cat | Paraná | 2020 | ON892996 | OP388788 | ON892916 | OR495668 |
| A001 | Cat | Federal Distrito | 2016 | ON892950 | OP388782 | ON892948 | OR495697 |
| 426 GAL GO | Human | Goiás | - | PX511341 | PX511353 | PX511365 | PX511377 |
| 431 GAL GO | Human | Goiás | - | PX511342 | PX511354 | PX511366 | PX511378 |
| Cat 1 ES | Cat | Espírito Santo | 2020 | ON892989 | OP388729 | ON892890 | OR495640 |
| Cat 3 ES | Cat | Espírito Santo | 2020 | ON893018 | OP388757 | ON892892 | OR495642 |
| Cat 5 ES | Cat | Espírito Santo | 2020 | ON892992 | OP388732 | ON892894 | OR495644 |
| Cat 1 MG | Cat | Minas Gerais | 2021 | ON893037 | OP388758 | ON892929 | OR495682 |
| Cat 2 MG | Cat | Minas Gerais | 2021 | ON893038 | OP388759 | ON892930 | OR495683 |
| Cat 3 MG | Cat | Minas Gerais | 2021 | ON893039 | OP388760 | ON892931 | OR495684 |
| Cat 4 MG | Cat | Minas Gerais | 2021 | ON893040 | OP388761 | ON892932 | OR495685 |
| Cat 5 MG | Cat | Minas Gerais | 2021 | ON893041 | OP388762 | ON892933 | OR495686 |
| Cat 6 MG | Cat | Minas Gerais | 2021 | ON893042 | OP388763 | ON892934 | OR495687 |
| 5153 SP | Cat | São Paulo | 2012 | ON893012 | OP388750 | ON892937 | OR495690 |
| 5522 SP | Cat | São Paulo | 2012 | ON893014 | OP388753 | ON892940 | OR495693 |
| 6235 SP | Cat | São Paulo | 2012 | ON893015 | OP388754 | ON892941 | OR495694 |
| 8128 SP | Cat | São Paulo | 2012 | ON893016 | OP388755 | ON892942 | OR495695 |
| 98 GAL | Cat | Pernambuco | 2016 | ON892981 | OP388721 | ON892882 | OR495632 |
| 99 GAL | Cat | Pernambuco | 2016 | ON892982 | OP388722 | ON892883 | OR495633 |
| 101 GAL | Cat | Pernambuco | 2016 | ON892983 | OP388723 | ON892884 | OR495634 |
| 102 GAL | Cat | Pernambuco | 2016 | ON892984 | OP388724 | ON892885 | OR495635 |
| 104 GAL | Cat | Pernambuco | 2016 | ON892986 | OP388726 | ON892887 | OR495637 |
| 105 GAL | Cat | Pernambuco | 2016 | ON892987 | OP388727 | ON892888 | OR495638 |
| 106 GAL | Cat | Pernambuco | 2016 | ON892988 | OP388728 | ON892889 | OR495639 |
| 2 PB | Cat | Paraíba | 2018/2020 | ON892997 | OP388736 | ON892917 | OR495669 |
| 6 PB | Cat | Paraíba | 2018/2020 | ON892998 | OP388737 | ON892918 | OR495670 |
| 9 PB | Cat | Paraíba | 2018/2020 | ON892999 | OP388738 | ON892919 | OR495671 |
| 18 PB | Cat | Paraíba | 2018/2020 | ON893000 | OP388739 | ON892920 | OR495672 |
| 21 PB | Cat | Paraíba | 2018/2020 | ON893001 | OP388740 | ON892921 | OR495673 |
| 26 PB | Cat | Paraíba | 2018/2020 | ON893002 | OP388741 | ON892922 | OR495674 |
| 35 PB | Cat | Paraíba | 2018/2020 | ON893004 | OP388743 | ON892924 | OR495675 |
| 38 PB | Cat | Paraíba | 2018/2020 | ON893005 | OP388744 | ON892925 | OR495676 |
| 40 PB | Cat | Paraíba | 2018/2020 | ON893006 | OP388745 | ON892926 | OR495677 |
| 46 PB | Cat | Paraíba | 2018/2020 | ON893008 | OP388747 | ON892928 | OR495679 |
| 441 GAL CE | Human | Ceará | - | PX511343 | PX511355 | PX511367 | PX511379 |
| 442 GAL CE | Human | Ceará | - | PX511344 | PX511356 | PX511368 | PX511380 |
| 443 GAL CE | Human | Ceará | - | PX511345 | PX511357 | PX511369 | PX511381 |
| 448 GAL CE | Human | Ceará | - | PX511346 | PX511358 | PX511370 | PX511382 |
| Isolates | **Sources** | **Geographic origin** | **Isolation year** | **AST 3SET** | **GPD1 2SET** | **CPLA 2 1SET** | **SPBR_05954** |
| 449 GAL CE | Human | Ceará | - | PX511347 | PX511359 | PX511371 | PX511383 |
| 6519 CE | Human | Ceará | - | PX511348 | PX511360 | PX511372 | PX511384 |
| 2240 CE | Human | Ceará | - | PX511349 | PX511361 | PX511373 | PX511385 |
| 7834 CE | Human | Ceará | - | PX511350 | PX511362 | PX511374 | PX511386 |
| CL | Human | Paraguay | - | ON892953 | OP388783 | ON892946 | OR495680 |
| CML | Human | Paraguay | - | ON892954 | OP388784 | ON892947 | OR495681 |
| DMIC 206254 | Human | Argentina | - | PX511351 | PX511363 | PX511375 | PX511387 |
| DMIC 216676 | Human | Argentina | - | PX511352 | PX511364 | PX511376 | PX511388 |

*AST*: Aspartate aminotransferase, *GPD1*: Glycerol-3-phosphate dehydrogenase fragment 2, *CPLA2*: Cytosolic phospholipase A2 and *SPBR_05954*: uncharacterized protein SPBR_05954. CFP: Coleção de Fungos Patogênicos/ Pathogenic Fungi Collection, Instituto Nacional de Infectologia Evandro Chagas, INI Fiocruz.
