# Supplementary material for: Susceptibility Profiles Among Sporothrix brasiliensis Genotypes in the Ongoing Zoonotic Sporotrichosis Epidemic in Brazil, Argentina and Paraguay
Source: Mycopathologia. 2026 Oct 15;191(6):89. doi: 10.1007/s11046-026-01113-1 (PMC13672573; doi:10.1007/s11046-026-01113-1)
Supplement: Supplementary file 2 [file 11046_2026_1113_MOESM2_ESM.docx]

Susceptibility Profiles Among *Sporothrix brasiliensis* Genotypes in the Ongoing Zoonotic Sporotrichosis Epidemic in Brazil, Argentina, and Paraguay

Mycopathologia

Vanessa Brito de Souza Rabello^1*^ ([0000-0002-9318-6240](https://orcid.org/0000-0002-9318-6240)), Marcus de Mello Teixeira^2^ ([0000-0002-3330-3847](https://orcid.org/0000-0002-3330-3847)), Fernando Almeida-Silva^1^ ([0000-0002-7378-1990](https://orcid.org/0000-0002-7378-1990)), Thiago Brasil de Moura Costa^1^ ([0009-0004-2448-3198](https://orcid.org/0009-0004-2448-3198)), Andrea Reis Bernardes-Engemann^1^ ([0000-0002-4997-8734](https://orcid.org/0000-0002-4997-8734)), Melissa Orzechowski Xavier^3^ ([0000-0002-3883-0080](https://orcid.org/0000-0002-3883-0080)), Rodrigo Almeida-Paes^1^ ([0000-0002-6639-6058](https://orcid.org/0000-0002-6639-6058)), María Cecilia López-Joffre^4^ ([0009-0006-1479-0615](https://orcid.org/0009-0006-1479-0615)), Cristina Elena Canteros^4^ ([0000-0002-0955-9340](https://orcid.org/0000-0002-0955-9340)), Lisandra Serra Damasceno^5^ ([0000-0003-1425-5912](https://orcid.org/0000-0003-1425-5912)), Isabella Dib Ferreira Gremião^6^ ([0000-0001-8428-7616](https://orcid.org/0000-0001-8428-7616)), José Guillermo Pereira Brunelli^7^ ([0000-0001-9660-4156](https://orcid.org/0000-0001-9660-4156)), Wieland Meyer^8^ ([0000-0001-9933-8340](https://orcid.org/0000-0001-9933-8340)), Rosely Maria Zancopé-Oliveira^1^ ([0000-0001-7619-1921](https://orcid.org/0000-0001-7619-1921))

1- Laboratório de Micologia, Instituto Nacional de Infectologia Evandro Chagas, Fundação Oswaldo Cruz, Rio de Janeiro, Brazil.

2- Núcleo de Medicina Tropical, Faculdade de Medicina, Universidade de Brasília (UnB), Brasília, Brazil.

3- Laboratório de Micologia, Faculdade de Medicina, Universidade Federal do Rio Grande (FAMED-FURG), Rio Grande do Sul (RS), Brazil.

4- Laboratorio Nacional de Referencia em Micología Clínica, Departamento de Micología. Instituto Nacional de Enfermedades Infecciosas INEI-ANLIS “ Dr. Carlos G. Malbrán, CABA, Argentina.

5- Hospital São José de Doenças Infecciosas (HSJ), Fortaleza, Ceará, Brazil.

6- Laboratório de Pesquisa Clínica em Dermatozoonoses em Animais Domésticos, Instituto Nacional de Infectologia Evandro Chagas, Fundação Oswaldo Cruz (Fiocruz), Rio de Janeiro, Brazil.

7- Centro de Especialidades Dermatologicas, Ministerio de Salud Pública y Bienestar Social, Paraguay.

8- Westerdijk Fungal Biodiversity Institute, Utrecht, Netherlands.

* Corresponding authors: [vanessabritorabello@gmail.com](mailto:vanessa.brito@ini.fiocruz.br) (Rabello, V.B.S^1^)

**Table Online Resource 2.** List of fungal strains studied, indicating their strain number, isolation source, geographical location, year of isolation, genotype and susceptibility profiles.

| Isolates | Sources | Geographic origin | Isolation year | Haplotype | MLST groups | Mating Type | Non-wild Type | Antifungal |
| --- | --- | --- | --- | --- | --- | --- | --- | --- |
| IPEC 41-1 | Cat | Rio de Janeiro | 1999 | H1 | I | MAT 2 | no |  |
| IPEC 41-2 | Cat | Rio de Janeiro | 1999 | H1 | I | MAT 2 | no |  |
| IPEC 47 | Cat | Rio de Janeiro | 1999 | H1 | I | MAT 2 | no |  |
| CFP 01256  (IPEC 98-2) | Cat | Rio de Janeiro | 1999 | H1 | I | MAT 2 | no |  |
| CFP 01257  (IPEC 98-5) | Cat | Rio de Janeiro | 1999 | H1 | I | MAT 2 | no |  |
| IPEC 856 | Cat | Rio de Janeiro | 2001 | H1 | I | MAT 2 | no |  |
| CFP 01254  (IPEC 15485-1) | Human | Rio de Janeiro | 1998 | H1 | I | MAT 2 | no |  |
| IPEC 15677-1 | Human | Rio de Janeiro | 1999 | H1 | I | MAT 2 | no |  |
| IPEC 16672 | Human | Rio de Janeiro | 1999 | H1 | I | MAT 2 | no |  |
| IPEC 17943 | Human | Rio de Janeiro | 2000 | H1 | I | MAT 2 | no |  |
| CFP 01255  (IPEC 19182) | Human | Rio de Janeiro | 2001 | H1 | I | MAT 2 | no |  |
| CFP 01022  (IPEC 19536) | Human | Rio de Janeiro | 2001 | H1 | I | MAT 2 | no |  |
| IPEC 19541 | Human | Rio de Janeiro | 2001 | H1 | I | MAT 2 | no |  |
| IPEC 19777 | Human | Rio de Janeiro | 2001 | H1 | I | MAT 2 | no |  |
| IPEC 19778 | Human | Rio de Janeiro | 2001 | H1 | I | MAT 2 | no |  |
| IPEC 28606 | Human | Rio de Janeiro | 2005 | H1 | I | MAT 2 | **yes** | **ITR** |
| IPEC 30771 | Human | Rio de Janeiro | 2006 | H1 | I | MAT 2 | no |  |
| IPEC 31047-1 | Human | Rio de Janeiro | 2006 | H1 | I | MAT 2 | no |  |
| IPEC 31091 | Human | Rio de Janeiro | 2007 | H1 | I | MAT 2 | no |  |
| IPEC 31515 | Human | Rio de Janeiro | 2007 | H1 | I | MAT 2 | no |  |
| IPEC 34180 | Human | Rio de Janeiro | 2008 | H1 | I | MAT 2 | **yes** | **ITR/AMB** |
| CFP 01043 | Environment | Rio de Janeiro | 2020 | H1 | I | MAT 2 | no |  |
| CFP 01042 | Cat | Rio de Janeiro | 2017 | H1 | I | MAT 2 | no |  |
| M716 | Cat | Rio Grande do Sul | 2012 | H3 | II | MAT 1 | no |  |
| M1023 | Cat | Rio Grande do Sul | 2012 | H3 | II | MAT 1 | no |  |
| M1078 | Cat | Rio Grande do Sul | 2012 | H3 | II | MAT 1 | no |  |
| M1079 | Human | Rio Grande do Sul | 2012 | H3 | II | MAT 1 | no |  |
| M1457 | Human | Rio Grande do Sul | 2013 | H3 | II | MAT 1 | no |  |
| M1543 | Cat | Rio Grande do Sul | 2013 | H3 | II | MAT 1 | no |  |
| M1547 | Dog | Rio Grande do Sul | 2013 | H3 | II | MAT 1 | no |  |
| M1564 | Humano | Rio Grande do Sul | 2013 | H3 | II | MAT 1 | no |  |
| M1620 | Dog | Rio Grande do Sul | 2013 | H3 | II | MAT 1 | no |  |
| M1878 | Human | Rio Grande do Sul | 2014 | H4 | III | MAT 1 | no |  |
| M2320 | Human | Rio Grande do Sul | 2014 | H3 | II | MAT 1 | no |  |
| M2444 | Human | Rio Grande do Sul | 2015 | H3 | II | MAT 1 | no |  |
| M2691 | Environment | Rio Grande do Sul | 2015 | H3 | II | MAT 1 | no |  |
| Isolates | **Sources** | **Geographic origin** | **Isolation year** | **Haplotype** | **MLST groups** | **Mating Type** | **Non-wild Type** | **Antifungal** |
| M3016 | Human | Rio Grande do Sul | 2015 | H3 | II | MAT 1 | no |  |
| M3357 | Human | Rio Grande do Sul | 2016 | H3 | II | MAT 1 | no |  |
| M3952 | Human | Rio Grande do Sul | 2017 | H3 | II | MAT 1 | **yes** | **ITR** |
| M4559 | Cat | Rio Grande do Sul | 2018 | H4 | III | MAT 1 | **yes** | **ITR** |
| M4720 | Cat | Rio Grande do Sul | 2018 | H3 | II | MAT 1 | no |  |
| M4748 | Cat | Rio Grande do Sul | 2018 | H3 | II | MAT 1 | no |  |
| M5150 | Human | Rio Grande do Sul | 2019 | H3 | II | MAT 1 | no |  |
| M6415 | Cat | Rio Grande do Sul | 2021 | H1 | I | MAT 1 | no |  |
| 1011 PR | Cat | Paraná | 2020 | H1 | I | MAT 2 | no |  |
| 1015 PR | Cat | Paraná | 2020 | H1 | I | MAT 2 | no |  |
| 1026 PR | Cat | Paraná | 2020 | H1 | I | MAT 2 | **yes** | **ITR/TER/AMB** |
| A001 | Cat | Federal Distrito | 2016 | H5 | III | MAT 2 | no |  |
| 426 GAL GO | Human | Goiás | - | H1 | I | MAT 2 | no |  |
| 431 GAL GO | Human | Goiás | - | H1 | I | MAT 2 | no |  |
| Cat 1 ES | Cat | Espírito Santo | 2020 | H1 | I | MAT 2 | no |  |
| Cat 3 ES | Cat | Espírito Santo | 2020 | H2 | II | MAT 1 | **yes** | **TER** |
| Cat 5 ES | Cat | Espírito Santo | 2020 | H1 | I | MAT 2 | no |  |
| Cat 1 MG | Cat | Minas Gerais | 2021 | H2 | II | MAT 1 | **yes** | **ITR/TER/AMB** |
| Cat 2 MG | Cat | Minas Gerais | 2021 | H2 | II | MAT 1 | **yes** | **ITR/TER/AMB** |
| Cat 3 MG | Cat | Minas Gerais | 2021 | H2 | II | MAT 1 | **yes** | **ITR/TER/AMB** |
| Cat 4 MG | Cat | Minas Gerais | 2021 | H2 | II | MAT 1 | **yes** | **ITR** |
| Cat 5 MG | Cat | Minas Gerais | 2021 | H2 | II | MAT 1 | no |  |
| Cat 6 MG | Cat | Minas Gerais | 2021 | H2 | II | MAT 1 | no |  |
| 5153 SP | Cat | São Paulo | 2012 | H1 | I | MAT 2 | no |  |
| 5522 SP | Cat | São Paulo | 2012 | H1 | I | MAT 2 | no |  |
| 6235 SP | Cat | São Paulo | 2012 | H1 | I | MAT 2 | no |  |
| 8128 SP | Cat | São Paulo | 2012 | H1 | I | MAT 2 | **yes** | **ITR/TER/AMB** |
| 98 GAL | Cat | Pernambuco | 2016 | H1 | I | MAT 2 | no |  |
| 99 GAL | Cat | Pernambuco | 2016 | H1 | I | MAT 2 | no |  |
| 101 GAL | Cat | Pernambuco | 2016 | H1 | I | MAT 2 | no |  |
| 102 GAL | Cat | Pernambuco | 2016 | H1 | I | MAT 2 | no |  |
| 104 GAL | Cat | Pernambuco | 2016 | H1 | I | MAT 2 | no |  |
| 105 GAL | Cat | Pernambuco | 2016 | H1 | I | MAT 2 | no |  |
| 106 GAL | Cat | Pernambuco | 2016 | H1 | I | MAT 2 | no |  |
| 2 PB | Cat | Paraíba | 2018/2020 | H1 | I | MAT 2 | no |  |
| 6 PB | Cat | Paraíba | 2018/2020 | H1 | I | MAT 2 | no |  |
| 9 PB | Cat | Paraíba | 2018/2020 | H1 | I | MAT 2 | no |  |
| 18 PB | Cat | Paraíba | 2018/2020 | H1 | I | MAT 2 | no |  |
| 21 PB | Cat | Paraíba | 2018/2020 | H1 | I | MAT 2 | no |  |
| 26 PB | Cat | Paraíba | 2018/2020 | H1 | I | MAT 2 | no |  |
| Isolates | **Sources** | **Geographic origin** | **Isolation year** | **Haplotype** | **MLST groups** | **Mating Type** | **Non-wild Type** | **Antifungal** |
| 35 PB | Cat | Paraíba | 2018/2020 | H1 | I | MAT 2 | no |  |
| 38 PB | Cat | Paraíba | 2018/2020 | H1 | I | MAT 2 | no |  |
| 40 PB | Cat | Paraíba | 2018/2020 | H1 | I | MAT 2 | no |  |
| 46 PB | Cat | Paraíba | 2018/2020 | H1 | I | MAT 2 | no |  |
| 441 GAL CE | Human | Ceará | - | H1 | I | MAT 2 | no |  |
| 442 GAL CE | Human | Ceará | - | H1 | I | MAT 2 | no |  |
| 443 GAL CE | Human | Ceará | - | H1 | I | MAT 2 | no |  |
| 448 GAL CE | Human | Ceará | - | H1 | I | MAT 2 | no |  |
| 449 GAL CE | Human | Ceará | - | H1 | I | MAT 2 | no |  |
| 6519 CE | Human | Ceará | - | H1 | I | MAT 2 | no |  |
| 2240 CE | Human | Ceará | - | H1 | I | MAT 2 | no |  |
| 7834 CE | Human | Ceará | - | H1 | I | MAT 2 | no |  |
| CL | Human | Paraguay | - | H6 | III | MAT 1 | no |  |
| CML | Human | Paraguay | - | H6 | III | MAT 1 | no |  |
| DMIC 206254 | Human | Argentina | - | H1 | I | MAT 2 | no |  |
| DMIC 216676 | Human | Argentina | - | H4 | III | MAT 1 | no |  |

Itraconazole (ITR), terbinafine (TER), amphotericin B (AMB).
